# Can an authentic EPortfolio succeed in First Year Medicine?

**DOI:** 10.15694/mep.2019.000236.1

**Published:** 2019-12-23

**Authors:** Iain Grom, Anna O'Neill

**Affiliations:** 1 University of Glasgow

**Keywords:** E portfolio, Authentic, First-Year, Medicine

## Abstract

This article was migrated. The article was not marked as recommended.

Introduction

Despite evidence supporting ePortfolio use to promote reflective practice, most published studies focus on its use in senior years. This study explored how well the introduction of the Undergraduate Medical ePortfolio (UMeP) in Year 1 of an early clinical contact curriculum met its aims of supporting reflective practice and introducing portfolio learning. Effective mentoring, organisational support, authenticity and adequate time are key to success.

Methods

A constructivist study heard the voices of students n=14 (2 focus groups) and tutors n=6 (semi-structured interviews) who had used the UMeP in its first introductory year. Thus, triangulation was employed to gain a deeper appreciation.

Results

Analysis uncovered four major themes- *Reflective Practice* **,** *Support/Training* **,** *ePortfolio functions,*and *Feedback/Assessment.* The study revealed support for the introduction of portfolio learning in Year 1 Medicine, for its role in promoting reflective practice and for maintaining formative assessment.

Discussion

EPortfolio introduction in Year 1 provided a valuable early introduction to reflection and life-long-learning habits. Regular small group tutor support and feedback were positive factors. The study revealed a need for tutor training on reflection feedback. Introduced at a stage without the weight of clinical commitments, this ePortfolio’s limited demands allowed students time to learn and become accustomed to its requirements. Scaffold boxes in ePortfolio forms facilitated reflective writing. Use of an authentic portfolio linked to a professional version was a key engagement factor. Through sharing submissions students learned from each other about reflective writing and confidentiality. Better curriculum integration is needed to develop its potential.

## Introduction

EPortfolios are used as repositories for evidence for continuing professional development ( Gordon and Campbell, 2013). They have wide use in undergraduate training ( Chertoff, *et al.*, 2015) and postgraduate training ( Tochel, *et al.*, 2011). Eportfolios provide the platform for provision of evidence required for annual appraisal leading to revalidation by the UK General Medical Council (NES). They may provide authentic assessment, accountability and a means to support critical reflection ( Cambridge, Cambridge and Yancey, 2009), important in the development of critical thinking ( Sandars, 2009).

Portfolios are widely used in undergraduate Medical Education but often only in later clinical years. The paper based Personal and Professional Development Reflective portfolio used in Year 1 and 2 Medicine was replaced by the Undergraduate Medical ePortfolio (UMeP) nhseportfolios (NES).This provided students with the opportunity to use an authentic portfolio similar to the Foundation EPortfolio ( Smith, *et al.*, 2014) used by most UK medical graduates in early training years.

Introduction of this undergraduate ePortfolio had the twin aims of introducing students to ePortfolio IT and providing them with a platform for their reflective writing. New templates, including a scaffold structure to aid reflective writing, were created. The portfolio was situated within tutor-mentored small group learning (Vocational Studies- Communication Skills, Clinical Skills, Professionalism, Medical Ethics, Science in Medicine) running throughout the academic year. As mentor support is recognized as a key element in portfolio success ( Erik W Driessen, Van Tartwijk, Overeem, Vermunt and Van Der Vleuten, 2005) the small group tutors were ideally placed to provide feedback to students on their reflections.

Students reflected on elements of their learning within their small group such as their communication skills learning, a “Thought- Provoking Event”, an ethical case, their course work assignment --and their experiences of small group learning. Tutors then provided feedback on the students’ submissions via the Eportfolio platform. To support their reflective writing, students are taught use of reflection theory using Gibbs Reflective Cycle ( Gibbs.G, 1988) from year one of their medical curriculum. While many students reflect on medical issues for their “Thought-Provoking Event”, this is not a requirement. The educational focus is good reflective writing.

EPortfolio assessment is formative rather than summative, however completion is a required element for curriculum progression. Electronic portfolio use offered the possibility of a quick response to reflective writing important in encouraging engagement ( Arntfield, *et al.*, 2016) together with a facility for students to store and build upon their reflections. On the other hand, the electronic format can pose a challenge ( Andrews and Cole, 2015); ( Birks, *et al.* 2016) to users, especially tutors, in adapting to the new technology.

## Methods

A qualitative descriptive study ( Creswell, 2014) was selected to hear the voices of participants who had one full academic years’ experience of using the new ePortfolio and gain insight into their thinking ( Barbour, 2007). Interview and focus group topic guides were prepared from a literature review and earlier course evaluation.

Fourteen students from a class of 240 were recruited through convenience sampling to two focus groups. The students, (nine male and five female) included UK, EU and International students, which was broadly representative of the medical class. Six tutors (two male and four female) from 25 possible participants were recruited, also through convenience sampling, to engage in semi-structured interviews. The tutors had a broad range of teaching experience, ranging from new to experienced, with many holding other educational roles such as G.P. Trainers, Appraisers and Educational Supervisors.

Focus group sessions and interviews were audio-recorded and transcribed. Member checking for accuracy did not result in any changes. Adjustments to topic guides were made according to responses received in an iterative manner. The transcripts were manually coded ( Creswell, 2014). This was independently verified through peer review. The codes were regrouped to form themes to assist in the formation of a narrative description of findings. Research ethics approval was received for the study.

## Results/Analysis

Four themes were identified from analysis of the coded material ( Creswell, 2014) - Reflective Practice, Support and Training, ePortfolio Functions and Feedback/Assessment.

### Reflective Practice

A prime function of this ePortfolio is to provide a platform for reflective writing that in turn fosters reflective practice. This study revealed that there was broad, though not universal, support for reflective practice:

*Reflective practice is an amazing tool and something that we should definitely be instilling in every medical professional.*(Student, focus group (FG) 1)

Early curriculum years students need encouragement and also to understand the reasons behind reflective writing ( Ryan, 2013). Their small group tutors described role models and provided students with examples from their own reflective writing

*..showing them my appraisal portfolio .. I’m doing this, so you might as well start now because you are going to be doing it [reflective writing] for the rest of your life.* (Tutor 4)

Unused to the art of reflective writing, students, for the most part, appreciated the provision of scaffold boxes which approximate to Gibbs reflective cycle ( Gibbs.G, 1988) in the ePortfolio forms:

*I quite like the text boxes because they like make you, go through the whole reflective thing* (Student, FG2)

Tutors also felt that the boxes provided good support for reflective writing:

*I think the students would ..give you half a paragraph if it was a blank page. I think they need prompts to realise what they’ve to focus on.* (Tutor 4)

The written reflections were considered by tutors to be of generally good, though variable, standard. Reflective writing produced by students studying for a second degree was reported to be of higher quality than that produced by students studying for their first degree. Tutors found that students were well engaged.

*..just saw these first years engaging in a way that was amazing..* (Tutor 6)

The quality of submission and engagement level and utility was indeed felt to be much better than the previous paper portfolio:

.. *feedback won’t get lost, they can always view it, they can see how that has been year to year, and use it as a part of their reflective training.* (Tutor 1)

Though UMeP IT does not facilitate sharing submissions with peers, tutors used the reflective writing in their small group work with good effect to demonstrate other perspectives

*..we shared our Thought-Provoking Events. The whole group .. gained some feedback, outsiders’ perspective towards it rather than just seeing a minimal sentence or two (of tutor feedback).*(Student, FG1)

Confidentiality issues that may pose a threat to reflective writing in ePortfolios were seen as a concern by participants.

*I .. would worry about .. disclosing it to the whole group.* (Student, focus group 1)

Although the shared IT system with the Foundation ePortfolio gave some reassurance about the safety of the system, early exposure to confidentiality issues through the UMeP would appear to be a benefit of ePortfolio introduction from Year 1.

Both timing and adequate time for completion and feedback were discussed. Students felt that, in Year 1, this ePortfolio was “ *not that extensive*” (Student, FG 1), and that they had adequate time to complete it.

On the other hand, tutors tended to feel that they required more time to provide effective feedback. Though students had adequate time there was still a tendency to leave submission until late in the final semester with little time for tutor feedback and increasing the possibility of inauthentic submission through time pressure. Both students and tutors did, nonetheless, agree on the merits of earlier timely submission:

*I think the best thing for reflective knowledge .. just to have a clear head and to be able to .. gather your thoughts..* (Student, FG 1)

Despite agreement on the value, it remained difficult to achieve timely submission.

### Support/Training

Tutors felt that they managed to learn to use the ePortfolio without specific training though neither students nor tutors were well informed about the ePortfolio’s storage functions:

*..if I knew about that function, I would’ve uploaded things..* (Student, FG2)

There was a similar situation with online support.

Reassuringly, there was some evidence of beneficial use of electronic storage in allowing comparison with earlier submissions indicating life-long learning.

*..when I was writing my small group work reflection for second year I went back and looked at my first year one and I saw that there was improvement.* (Student, FG2)

There were also concerns among tutors about their ability to support reflection:

*I’m not sure I’m even reflecting correctly so I think probably the art of reflection could be better taught but it would probably need to be taught to tutors first.* (Tutor 4).

Students also saw a need for more educational backup. A successful ePortfolio needs educational support. Unfortunately, students had encountered some negative attitudes from some of their lecturers.

*..some of the lectures we go to were quite negative.*(Student, FG1)

Indeed, there was a perception among students that some other parts of the School of Medicine were not very engaged with the UMeP. In contrast, positive attitudes were expressed about the early years’ introduction of the ePortfolio as a preparation for professional practice:

*..it’s vital to get them in at first year, to get them used to making a portfolio, they will have to do it all of their professional life.* (Tutor 4)

Students also recognized the value of the early introduction of the UMeP:

*..that’s kind of what we are going to be doing later on aren’t we, we are going to be..making a habit ..as junior doctors, just building our portfolio and our personal development.*.(Student, FG1)

### Portfolio functions

This ePortfolio did also have its issues:

*..it looks like it was programmed a very long time ago by the kind of layout and how the menus work I.. guess we are bit spoiled .. from Facebook.* (Student, FG1)

### Assessment

At present, in the featured curriculum, portfolio assessment is formative. Evidence of adequate ePortfolio engagement is, however, required for progress. There were mixed feelings about possible summative assessment, the majority favouring continuation of formative assessment. Some felt that the introduction of summative assessment might stifle reflective thought making it just another task:

*It then becomes a task that you need to do and excel at and dehumanises it, marking that sort of work is far too subjective.* (Student, FG1)

Whilst others reported summative assessment may be a stimulus. Participants felt that their reflective writing in the UMeP could be difficult to assess requiring the setting and publication of agreed standards.

### Feedback

Feedback is recognized to be a key support element of reflective writing; the study found that students felt this to be beneficial:

.. *some are really descriptive and they actually write a lot and .. it really helps the person in their development, whereas just “good” is not very helpful.* (Student, FG1)

The quality of feedback appears to be a training issue. Knowledge of student feedback experience may serve to drive up standards.

## Discussion

This study found that an authentic ePortfolio linked to a professional portfolio used by medical graduates was an incentive, mirroring the findings of Belcher’s clinical years’ ePortfolio ( Belcher, *et al.*, 2014). EPortfolio authenticity was also found to be positive factor for students in Bleasel’s ( Bleasel, *et al.*, 2016) Australian Medical Schools study.

Whereas the students in Belcher’s study felt that clinical commitments competed with the time they had for their ePortfolio. This study suggests that this UMeP did not make onerous demands on students. Our Year 1 students did not suffer from the same time problem. It may be argued that the less demanding portfolio allows students time to learn about reflective writing and ePortfolio functions. Therefore, supporting the case for early curriculum introduction.

In common with Austin and Braidman’s initial years’ ePortfolio findings, tutor assistance was needed to promote reflective writing to students unfamiliar with the concept. Guidance is seen as key in producing effective reflection ( Ryan, 2013).

Our tutors commented that more mature students appear to produce better reflective writing. This accords with research findings that reflective capacity develops with maturity ( King and Kitchener, 2004). This being the case, a later years ePortfolio introduction might seem more appropriate, however our participants were found to be in support of an early portfolio introduction as a life-long-learning /Reflective practice initiation. Early introduction accords with Driessen’s recommendations ( Erik Driessen, van Tartwijk, Vermunt and van der Vleuten, 2003) and also with King and Kitchener’s ( King and Kitchener, 2004) finding that reflective thinking is associated with participation in educational programmes.

Adaptation of UMeP forms (Example - Appendix A) for use by Year 1 students supported their reflective writing with the aid of scaffold boxes in common with findings reported by Aronson ( Aronson, 2011). A blank un-scaffolded form was created for those who preferred to write unconstrained in response to student suggestions. Thus providing some student input and flexibility in UMeP provision. These portfolio features meet with recommendations for success ( Van Tartwijk and Driessen, 2009).

This study highlighted the support of enthusiastic and able small group mentors. These mentors had good knowledge of their students and continuing weekly contact throughout the academic year. This plays a positive role in fostering reflective practice through the UMeP. Therefore the necessary quick feedback on reflective writing found by Arntfield ( Arntfield, *et al.* 2016) is made more possible through the UMeP. Thus the key role of mentors in supporting an ePortfolio as in other studies is confirmed. In addition, students may be more confident in revealing their reflective thoughts to tutors they know well. An ePortfolio introduced only in clinical years is unlikely to benefit from this degree of mentor support.

The UMeP was situated within stable small group learning sets. Students were able to support each other with their approach to learning about the IT and reflective writing, demonstrating a positive application of social learning theory ( Mann, 1994). Sharing reflections is good educational practice ( Hall, *et al.*, 2012). It is also an important aspect in gaining educational value from reflective practice ( Brookfield, 2017); ( Siporin, 2013).

Though tutors had experience of reflective writing through their own appraisal portfolios, this study suggests a need for additional training in feedback on reflection. This may be met through providing structure for feedback using a Reflective writing rubric such as that provided by Hatton and Smith ( Hatton and Smith, 1995) together with feedback guidelines.

Through reading students submissions tutors were able to gain a greater insight into their students’ learning and also their own teaching fuelling their enjoyment of their small group teaching, echoing Buckley’s findings ( Buckley, *et al.*, 2009).

This study suggests that this UMeP did not make onerous demands on students. This may have allowed them more time to become acquainted with its IT and also the practice of reflective writing. Thus supporting its early curriculum introduction.

The study also revealed that students valued considered feedback, ideally backed up by e to one discussion, mirroring the findings of Arntfield ( Arntfield, *et al.*, 2016). Time available for completion and timing of submission was highlighted as potentially important. Despite the desire for feedback, students still had a tendency to make late submissions even when earlier deadlines had been agreed. This reduced the possibility of tutors providing feedback that students could effectively use ( Moores and Parks, 2010). Nonetheless, inauthentic submission due to time pressure was a fairly prominent discussion feature of both student focus groups, in common with previous work ( Birden and Usherwood, 2013).

Through shared small group discussion of ePortfolio reflections students were able to learn and appreciate confidentiality issues in a non-threatening environment. Thus, they may be better prepared to manage more challenging clinical reflections. In addition an opportunity can be taken to discuss the issues associated with junior doctor, Hadiza Bawa-Garba, whose written reflective notes may have been used in legal proceedings ( Iacobucci, 2018); ( Bradshaw, 2018). Indeed reflective writing in the ePortfolio can provide an opportunity to discuss the updated GMC advice on what to include in written reflection ( GMC, 2018).

IT issues often prove to be a significant barrier to ePortfolio adoption ( Duque, *et al.*, 2006). Though participants in this study had some issues with the IT supporting this ePortfolio, this did not prove to be a great obstacle despite only minimal training. Perhaps this was due to its less onerous demands.

Students did report that they had encountered negative attitudes towards the ePortfolio in their lectures but did not say why. Belcher’s study ( Belcher, *et al.*, 2014) also revealed similar problems with their clinical years’ ePortfolio where negative IT attitudes were encountered among clinical staff. Some resistance to innovation might be expected in a busy curriculum with competition for students’ time and attention. Moreover, the educational value of reflection itself is not universally acknowledged ( Sandars, 2009). In order to overcome this, greater advocacy for reflection and the use of the UMeP ( Andrews and Cole, 2015) is required. In addition, the ePortfolio might have better support with better curriculum integration ( Chatham-Carpenter, Seawel and Raschig, 2010). Their review of Higher Education Institutions found top-down support is needed to establish clarity of purpose and course integration. An increased role in providing support for self-directed learning as reported by Beckers ( Beckers, Dolmans and van Merrienboer, 2016) may go some way to achieving this goal.

Summative assessment has, in the past, been suggested as prerequisite for portfolio success ( Erik W Driessen, Van Tartwijk, Overeem, Vermunt and Van Der Vleuten, 2005). It may also discourage portfolio use ( Pearson and Heywood, 2004). The UMeP, however, employed formative assessment. Course evaluation out with this study showed engagement levels in excess of 90%. Study participants saw a tension between learning and assessment similar to those of Sandars ( Sandars, 2009), most preferring formative assessment. As an early curriculum ePortfolio with an emphasis on learning this may be entirely appropriate.

### Strengths, Limitations and Future Direction

This study employed triangulation ( Creswell, 2014) whereby views of students and tutors can confirm or disconfirm each other to provide a more complete picture of participant views. It was conducted by a researcher closely involved with the ePortfolio within this context with good practical knowledge. This insider role adds strength to the study ( Kvale, 1996). Virtual saturation was achieved with the resources available. Though much effort was expended in recruiting more focus group participants and tutors, this did not prove possible within the time available.

The study looked at the experience of one cohort of students and tutors over one year in one institution with one adaptation of the UMeP. However, due to the rigour of this study, its findings may be transferable to other contexts. EPortfolio studies reported in literature often have similar context specific constraints. Nonetheless, the study findings reported may be of assistance to anyone considering an early year’s ePortfolio. This qualitative study was only able to sample the views of a convenience sample of focus group members and tutor interviewees, however as participants communicate freely with their colleagues their views are potentially also expressed in the study findings. This study was not able to analyse the reflective writing itself, nor the reflective writing feedback given by tutors. Further study of these elements of the ePortfolio experience following enhanced tutor feedback training would be an interesting future direction.

## Conclusion

This study suggests that an ePortfolio can be introduced with success early in the medical curriculum to support reflective practice. Contextual adaptation of the Undergraduate Medical ePortfolio as an authentic ePortfolio with its association to the postgraduate Foundation ePortfolio assisted in promoting its aims. Early ePortfolio introduction of a less demanding portfolio in a less busy clinical curriculum allows students additional time to learn together with the support of small group mentors to promote reflective practice and provide effective feedback. Students can learn key aspects of reflective writing such as confidentiality and what to include within a supportive cooperative environment. More senior students may have a better capacity to reflect but may not have the time or support. Good levels of engagement can be achieved with student input and formative assessment. Tutor training on reflection feedback may be required. As with many ePortfolios continued advocacy and better curriculum integration are needed.

## Take Home Messages

• Use of an authentic ePortfolio based on a format used in postgraduate years enhanced its support among early curriculum students
• Early curriculum introduction allows time for students to learn key points together
• Use of the ePortfolio in a small group teaching context aided by tutors who feedback on their own students’ reflections is a positive feature
• There is a need for further tutor training on reflection
• Institutional support for reflective practice and the role of an ePortfolio with good curriculum integration is important

## Notes On Contributors

Dr Iain Grom is Clinical Lecturer and Vocational Studies Tutor in Undergraduate Medicine at the University of Glasgow, UK. Interests - Undergraduate Medical Education, Learning and Teaching. ORCID: https://orcid.org/0000-0002-5564-2401

Dr Anna O’Neill is Senior Lecturer in Nursing and Health Care at the University of Glasgow, UK. ORCID: https://orcid.org/0000-0001-7843-9011

## Appendices

### Appendix A

Structured Reflection (Scaffold) Form example

## References

[ref1] Andrews T. and Cole C. (2015) Two steps forward, one step back: The intricacies of engaging with eportfolios in nursing undergraduate education. Nurse Education Today. 35(4), pp.568–572. 10.1016/j.nedt.2014.12.011 25656080

[ref2] Arntfield S. Parlett B. Meston C. N. Apramian T. (2016) A model of engagement in reflective writing-based portfolios: Interactions between points of vulnerability and acts of adaptability. Medical Teacher. 38(2), pp.196–205. 10.3109/0142159X.2015.1009426 25697109

[ref3] Aronson L. (2011) Twelve tips for teaching reflection at all levels of medical education. Medical Teacher. 33(3), pp.200–205. 10.3109/0142159X.2010.507714 20874014

[ref4] Barbour R. (2007) Doing Focus Groups. London: SAGE. 10.4135/9781849208956

[ref5] Beckers J. Dolmans D. and van Merrienboer J. (2016) e-Portfolios enhancing students’ self-directed learning: A systematic review of influencing factors. Australasian Journal of Educational Technology. 32(2), pp.32–46. 10.14742/ajet.2528

[ref6] Belcher R. Jones A. Smith L.-J. Vincent T. (2014) Qualitative study of the impact of an authentic electronic portfolio in undergraduate medical education. BMC Medical Education. 14, pp.265–265. 10.1186/s12909-014-0265-2 25515320 PMC4272766

[ref7] Birden H. H. and Usherwood T. (2013) “They liked it if you said you cried” how medical students perceive the teaching of professionalism. The Medical Journal of Australia. 199(6), pp.406–409. 10.5694/mja12.11827 24033214

[ref8] Birks M. Hartin P. Woods C. Emmanuel E. (2016) Students’ perceptions of the use of eportfolios in nursing and midwifery education. Nurse Education in Practice. 18, pp.46–51. 10.1016/j.nepr.2016.03.003 27235565

[ref9] Bleasel J. Burgess A. Weeks R. and Haq I. (2016) Feedback using an ePortfolio for medicine long cases: quality not quantity. BMC Medical Education. 16(1), p.278. 10.1186/s12909-016-0801-3 27769240 PMC5073895

[ref10] Bradshaw D. P. (2018) E-portfolios, reflections and the case of Dr Bawa-Garba. British Journal of Hospital Medicine,79(3), pp.126–127. 10.12968/hmed.2018.79.3.126 29528747

[ref11] Brookfield S. (2017) Becoming a Critically Reflective Teacher. Jossey-Bass(Downloaded: 4 October 2019).

[ref12] Buckley S. Coleman J. Davison I. Khan K. S. , (2009) The educational effects of portfolios on undergraduate student learning: A Best Evidence Medical Education (BEME) systematic review. BEME Guide No. 11. Medical Teacher. 31(4), pp.282–298. 10.1080/01421590902889897 19404891

[ref13] Cambridge D. Cambridge B. and Yancey K. (2009) Electronic Portfolios 2.0 Emergent Research on Implementation and Impact. Sterling, Virginia: Stylus.

[ref14] Chatham-Carpenter A. Seawel L. and Raschig J. (2010) Avoiding the Pitfalls: Current Practices and Recommendations for ePortfolios in Higher Education. Journal of Educational Technology Systems. 38(4), pp.437–456. 10.2190/ET.38.4.e

[ref15] Chertoff J. Wright A. Novak M. Fantone J. (2015) Status of portfolios in undergraduate medical education in the LCME accredited US medical school. Medical Teacher.pp.1–11. 10.3109/0142159X.2015.1114595 26652913

[ref16] Creswell J. W. (2014) Research Design. 4th edn. Thousand Oaks, California: Sage.

[ref17] Driessen E. van Tartwijk J. Vermunt J. and van der Vleuten C. (2003) Use of portfolios in early undergraduate medical training. Medical Teacher. 25(1), pp.18–23. 14741854 10.1080/0142159021000061378

[ref18] Driessen E. W. Van Tartwijk J. Overeem K. Vermunt J. D. , (2005) Conditions for successful reflective use of portfolios in undergraduate medical education. Medical Education. 39(12), pp.1230–1235. 10.1111/j.1365-2929.2005.02337.x 16313582

[ref19] Duque G. Finkelstein A. Roberts A. Tabatabai D. , (2006) Learning while evaluating: the use of an electronic evaluation portfolio in a geriatric medicine clerkship. BMC Medical Education. 6, pp.4–4. 10.1186/1472-6920-6-4 16409640 PMC1361794

[ref20] Gibbs G,(1988) Reflective Learning Cycle. Available at: http://www.cumbria.ac.uk/Public/LISS/Documents/skillsatcumbria/ReflectiveCycleGibbs.pdf( Accessed: 21/01/16).

[ref21] GMC(2018) The Reflective Practitioner. Available at: https://www.gmc-uk.org/education/standards-guidance-and-curricula/guidance/reflective-practice( Accessed: 29 September 2019).

[ref22] Gordon J. A. and Campbell C. M. (2013) The role of ePortfolios in supporting continuing professional development in practice. Medical Teacher. 35(4), pp.287–294. 10.3109/0142159X.2013.773395 23590423

[ref23] Hall P. Byszewski A. Sutherland S. and Stodel E. J. (2012) Developing a Sustainable Electronic Portfolio (ePortfolio) Program That Fosters Reflective Practice and Incorporates CanMEDS Competencies Into the Undergraduate Medical Curriculum. Academic Medicine. 87(6), pp.744–751. 10.1097/ACM.0b013e318253dacd 22534601

[ref24] Hatton N. and Smith D. (1995) Reflection in teacher education: Towards definition and implementation. Teaching and Teacher Education,11(1), pp.33–49. 10.1016/0742-051x(94)00012-u

[ref25] Iacobucci G. (2018) New guidance on reflective practice to be published in wake of Bawa-Garba case’, Bmj-British Medical Journal, 361. 10.1136/bmj.k2225 29776971

[ref26] King P. M. and Kitchener K. S. (2004) Reflective Judgment: Theory and Research on the Development of Epistemic Assumptions Through Adulthood. Educational Psychologist. 39(1), pp.5–18. 10.1207/s15326985ep3901_2

[ref27] Kvale S. (1996) InterViews An Introduction to Qualtative Research Interviewing. Thousand Oaks: SAGE.

[ref28] Mann K. V. (1994) Educating medical students: lessons from research in continuing education. Academic Medicine. 69(1), pp.41–47. 10.1097/00001888-199401000-00013 8285999

[ref29] Moores A. and Parks M. (2010) Twelve tips for introducing E-Portfolios with undergraduate students. Medical Teacher. 32(1), pp.46–49. 10.3109/01421590903434151 20095774

[ref30] NHS e-portfolios. Available at: nhseportfolios.org( Accessed: 5 October 2019).

[ref31] Pearson D. J. and Heywood P. (2004) Portfolio use in general practice vocational training: a survey of GP registrars. Medical Education. 38(1), pp.87–95. 10.1111/j.1365-2923.2004.01737.x 14962030

[ref32] Ryan M. (2013) The Pedagogical Balancing Act: Teaching Reflection in Higher Education. Teaching in Higher Education,18(2), pp.144–155. 10.1080/13562517.2012.694104

[ref33] Sandars J. (2009) The use of reflection in medical education: AMEE Guide No. 44. Medical Teacher. 31(8), pp.685–695. 10.1080/01421590903050374 19811204

[ref34] Siporin S. (2013) Using Eportfolios to Aid Reflection in Introductory Psychology. Psychology Learning & Teaching,12(1), pp.71–75. 10.2304/plat.2013.12.1.71

[ref35] Smith L.-J. Belcher R. Coppola W. Gill D. (2014) Successful collaboration in education: the UMeP. The Clinical Teacher. 11(7), pp.546–550. 10.1111/tct.12212 25417985

[ref36] Tochel C. Beggs K. Haig A. Roberts J. (2011) Use of web based systems to support postgraduate medical education. Postgraduate Medical Journal. 87(1034), pp.800–806. 10.1136/postgradmedj-2011-130007 21984741

[ref37] Van Tartwijk J. and Driessen E. W. (2009) Portfolios for assessment and learning: AMEE Guide no. 45. Medical Teacher. 31(9), pp.790–801. 10.1080/01421590903139201 19811183

