# Supplementary material for: Can an authentic EPortfolio succeed in First Year Medicine?
Source: MedEdPublish (2016). 2019 Dec 23;8:236. [Version 1] doi: 10.15694/mep.2019.000236.1 (PMC10712622; doi:10.15694/mep.2019.000236.1)
Supplement: Peer review history

# Reviewer report: Reviewer response for version 1

**DOI:** 10.21956/mep.19001.r27256

**Published:** 2020-01-29

**Authors:** Tharin Phenwan

**Affiliations:** 1 University of Dundee

This review has been migrated. The reviewer awarded 3 stars out of 5 This article provided the results of introducing ePortfolio to students that encouraged them to practise reflective writing. The formative approach of this activity seems suitable and practical for early year students, along with Gibbs reflective framework to guide them along with tutors’ support.Introduction-The background introduction part was good and relevant but the aims and current gaps that motivated this work were still unclear. Was it to assess the practicability of ePortfolio? Or would it be better to introduce RP to students in their early year? Could you clarify them?-Did they write about their experiences in a particular module of their study? Or was the ePortfolio opened for the whole year and they could write about any memorable event for them?Methods-Recruitment: how did you recruit them? (emails/posters/websites/ etc?)-Were there any steps during the recruitment process to ensure that the students were not forced or coerced to join? Or to ensure that there would be no COI.-The methodology section is still a bit unclear. Which approach that you use for this work? In the abstract, there was a mention of triangulation but did you mean by investigator or methodology or something else?Results-RP theme: were there any significant differences in students’ writing between mature students and non-mature ones? E.g. different stage of Gibb’s cycle. -Was there any improvement of their writing during the year e.g. a shift from the early stage of the cycle to the advanced ones? Since the tutors gave feedback that the writings were generally good with variable standard, you may need to expand this part further.-The other challenge here on writing practice is how do we know that the students truly reflect on their experiences instead of writing down the buzz words that sound ad look reflective i.e. become a reflective zombie? (Please see this article https://www.ncbi.nlm.nih.gov/pubmed/30353284)-Support/training: was there any support for tutors for giving feedback as well?-Portfolio and feedback theme: these themes may need more expansion. From the current results, it showed that there was an issue about the programme and the feedback quality; why is that? How did it happen that way? How did it affect their learning experiences? What is needed to be done? etcDiscussion-The last paragraph on page 6 was a bit unclear. Did they write confidential issues on their ePortfolio? Or did you mean that by using ePortfolio, it helped them learn about this?Strengths/limitations-The first sentences about triangulation may be under the methodological section to help readers understand the method better. This article will be of interest to medical educators and other healthcare professionals who are involved with portfolio, feedbacks, and undergraduate training.

Reviewer Expertise:

NA

No decision status is available

# Reviewer report: Reviewer response for version 1

**DOI:** 10.21956/mep.19001.r27255

**Published:** 2020-01-06

**Authors:** Megan Anakin

**Affiliations:** 1 University of Otago

This review has been migrated. The reviewer awarded 3 stars out of 5 Thank you for submitting this article about introducing eportfolios in the introductory year of medical education. This article caught my interest because at my medical school, we are wondering how we can better promote critical reflection with our students at all stages of their medical education. The idea of introducing eportfolios early in the medical programme can be a way to support students as they develop reflective writing and IT skills. The findings of this study are summarised in great detail in this article. The authors may wish to consider providing a greater amount of detail in their outline of the research territory in the introduction and especially more detail in the methods section to help the reader better understand the procedures used to collect and analyse the data in this study. The authors may wish to consider enhancing the description of the aim of the study at the end of the introduction or introducing the research questions guiding the study design at the beginning methods section to help the reader better understand the intentions and choices made by the researchers of this study. Currently, the aims described in the abstract and in the third paragraph of the introduction are about introducing the ePortfolio as a teaching/learning tool rather than revealing the purpose of this study to the reader. To enhance understanding the results, the authors may wish to more fully describe the questions that they asked students and tutors so the reader can understand how similar they were. Likewise, an explanation of how responses to the questions were analysed would help the reader understand when and how codes of the data sets from the students and tutors were combined to produce the final set of themes. The section title ‘results/analysis’ is confusing. The authors may wish to consider further describing their data analysis procedures in the methods section and clearly signpost that they are reporting results in the results section. The authors may wish to consider improving how the results are reported by further introducing and organising the results within each theme further. For example, within the theme of reflective practice, fostering reflective practice was presented first but then followed up with additional dimensions such as quality of reflective writing, confidentiality, and timing for completion and feedback. This organisation and signposting will help the reader make sense of the lengthy discussion section and better appreciate the links the authors make between the results and discussion sections. The third theme only has one sentence and quotation to support it. The authors may wish to consider providing further evidence to support this theme, otherwise, the third theme does not make sense as it is currently presented to the reader. At the beginning of the results section, the authors signpost that four themes were identified, however, there are five headings used to report the results. The authors may wish to combine the assessment and feedback themes as indicated at the beginning of the results section to help to reinforce the expectations they set for the reader. To strengthen the assessment/feedback theme, the authors may wish to add quotations from tutors to provide a balance of views from both students and tutors, if not, then this theme should be presented as originating from students only or predominantly. To enhance the discussion section, the authors may wish to consider placing the statements in the first paragraph might after they summarise the key findings of the study in relation to its aim (or the central research question that was investigated). Acting on suggestions like these will help the reader to better understand the merit of this article and to interpret the significance of the results. Even though I have made several suggestions for improvement, I feel that this article addresses a topic that will be of interest to medical educators and other healthcare professionals.

Reviewer Expertise:

NA

No decision status is available
